# Investigating the utility of Google trends for Zika and Chikungunya surveillance in Venezuela

**DOI:** 10.1186/s12889-020-09059-9

**Published:** 2020-06-16

**Authors:** Ricardo Strauss, Eva Lorenz, Kaja Kristensen, Daniel Eibach, Jaime Torres, Jürgen May, Julio Castro

**Affiliations:** 1 Bernhard Nocht Institute for Tropical Medicine, Research Group Infectious Disease Epidemiology, Hamburg, Germany; 2 Institute of Medical Biostatistics, Epidemiology and Informatics, University Medical Center, Mainz, Germany; 3 Faculty of Life Sciences, Hamburg University of Applied Sciences, Ulmenliet 20, 21033 Hamburg, Germany; 4 Instituto de Medicina Tropical, Universidad Central de Venezuela, Caracas, Venezuela

**Keywords:** Surveillance systems, Chikungunya, Zika, Internet search queries

## Abstract

**Introduction:**

Chikungunya and Zika Virus are vector-borne diseases responsible for a substantial disease burden in the Americas. Between 2013 and 2016, no cases of Chikungunya or Zika Virus were reported by the Venezuelan Ministry of Health. However, peaks of undiagnosed fever cases have been observed during the same period. In the context of scarce data, alternative surveillance methods are needed. Assuming that unusual peaks of acute fever cases correspond to the incidences of both diseases, this study aims to evaluate the use of Google Trends as an indicator of the epidemic behavior of Chikungunya and Zika.

**Methods:**

Time-series cross-correlations of acute fever cases reported by the Venezuelan Ministry of Health and data on Google search queries related to Chikungunya and Zika were calculated.

**Results:**

A temporal distinction has been made so that acute febrile cases occurring between 25th of June 2014 and 23rd of April 2015 were attributed to the Chikungunya virus, while cases occurring between 30th of April 2015 and 29th of April 2016 were ascribed to the Zika virus. The highest cross-correlations for each disease were shown at a lag of 0 (*r* = 0.784) for Chikungunya and at + 1 (*r* = 0.754) for Zika.

**Conclusion:**

The strong positive correlation between Google search queries and official data on acute febrile cases suggests that this resource can be used as an indicator of endemic urban arboviruses activity. In the Venezuelan context, Internet search queries might help to overcome some of the gaps that exist in the national surveillance system.

## Background

Chikungunya and Zika are vector-borne viral infections. The primary vectors are the mosquito species *Aedes aegypti* and *Aedes albopictus* [1]. Although the Zika virus infection is asymptomatic in the majority of cases [1], the disease may cause fever, bodily and joint pain and fatigue amongst other symptoms [2]. In February 2016, the World Health Organization (WHO) declared Zika a Public Health Emergency of International Concern due to its association with microcephaly and the Guillain-Barré Syndrome [3]. The cumulative Zika cases reported to the Pan American Health Organization (PAHO) between 2015 and December 2017 accounted for 535,000 cases [4]. In Venezuela, the first autochthonous vector-borne Zika case was reported in November 2015 [5].

Chikungunya virus infection is symptomatic in 85% of infected individuals, usually presenting with high fever, rash, headache, and polyarthritis that may progress into a chronic condition in up to 50–60% of cases [1, 6]. During 2013 and 2014, more than one million suspected cases of Chikungunya were reported to PAHO [7]. The first case of imported Chikungunya in Venezuela was officially reported in June 2014 [8].

Coinciding with the Zika and Chikungunya outbreak periods, peaks of acute fever cases of unknown origin have been reported by the official epidemic bulletins in Venezuela [9–11]. In this setting with limited laboratory capacities, it is unknown to which extent Zika virus or Chikungunya virus infections have contributed to these peaks of fever cases.

Communicable Disease Surveillance Systems (CDSS) aim to facilitate quick response to disease occurrence and to prioritize the allocation of resources [12, 13]. CDSSs in developing countries are challenged by the lack of modern technology, lack of integrated reporting systems, and political uncertainties [14]. Furthermore, due to limited resources, CDSSs in developing countries may face problems in providing data in a timely manner endangering adequate detection and delaying the response, particularly relevant in the context of rapidly spreading diseases [15, 16]. Moreover, the ongoing economic and political crisis has led to a humanitarian and migration emergency as well as a collapse of the Venezuelan health system, simultaneously deteriorating the national surveillance, allowing the spread of various emerging infectious diseases to neighboring countries [17–19].

Meanwhile, as global internet use has increased, web searches have become a common source for health information [20]. Web searches are not only a valuable resource for the individual who seeks health information, but also for the scientific community, as search queries may contain geographic and timely information about disease outbreaks [21]. Harnessing data generated by web searches can enhance surveillance systems by providing real-team proxy data thus enabling faster responses [22].

To analyze search trends, Google has developed a tool named Google Trends that monitors searches [22]. An analysis of 64 different diseases has shown that vector-borne diseases, among others, are well suited for web search based surveillance [23]. Moreover, Google search queries of different vector-borne diseases, mainly Dengue virus, have been shown by us and others to correlate well with the number of cases captured by surveillance systems in various countries, including Venezuela [15, 24–26].

This study aims to evaluate the utility of Google Trends as an indicator of the epidemic behavior of Zika and Chikungunya in a context of sparse data availability. It is assumed that an unusual accumulation of acute fever cases reported by the Ministry of Health (MoH) correspond to actual outbreaks of these diseases.

## Materials and methods

### Data sources

#### Officially reported cases

Weekly incidence data on reported acute fever cases in Venezuela from January 2014 to April 2016 were obtained from official reports available at the Weekly Epidemiological Report of the Venezuelan MoH (Boletín Epidemiológico Semanal). “Expected cases” according to the MoH, were calculated based on data of the previous years as a projection used to design public health interventions. The expected cases correspond to official data that were publicly available in the epidemiologic bulletins under the category “fever of unspecific diagnosis”. Variations in the incidence curve of less than 2 standard deviations of the last 5 years mean represented the7 expected cases. They were used as a proxy indicator of endemic, or expected, fever cases, assuming that any modification of this pattern indicates an unusual activity.

#### Google trends and query terms

Weekly data on Internet searches for Chikungunya and Zika was obtained from Google Trends (https://trends.google.com/trends/) on 30th of January 2019. Google Trends provides a time series index of the volume of queries that users enter into Google in a given geographic area during a given period of time. This tool allows for the systematic collection of information on all searches related to a given keyword (or list of keywords) and related terms.

We accessed trends.google.com and separately entered the terms “Zika” and “Chikungunya”. Then, we customized the search for Venezuela in a timeframe matching the relevant period from 2014 to 2016. Google trends automatically clusters queries in Spanish language related and/or similar to the terms “Zika” and “Chikungunya”. Isolated terms related to particular symptoms such as “fever” were not included to avoid overlap with queries for other diseases. Next, we downloaded the data in csv. Format used to produce the google trends curves. This data was reproduced in a relative scale from 0 to 100, with 0 representing no related queries and 100 the maximum number of related queries.

### Data analysis / statistical analyses

Statistical analyses were conducted using R version 3.4.2. Line plots and cross-correlations were applied to assess the association between Google search trends and the officially reported febrile cases.

Cross-correlations are used to characterize time dependence between time-series from two different data sources. Both time series were normalized in order to account for varying units and scales. The time dependence between two time-series is termed as lag, which indicates the degree and direction of associations. A lag of − 1 suggests that a peak in Google trends precedes a peak in the officially reported febrile cases by 1 week, and vice versa for positive lags. Considering the objectives of this study, a positive association between the two time-series supports the usefulness of Google Trends as a supportive tool for timely outbreak detection/reporting.

## Results

### Time trend

**Fig. 1:**
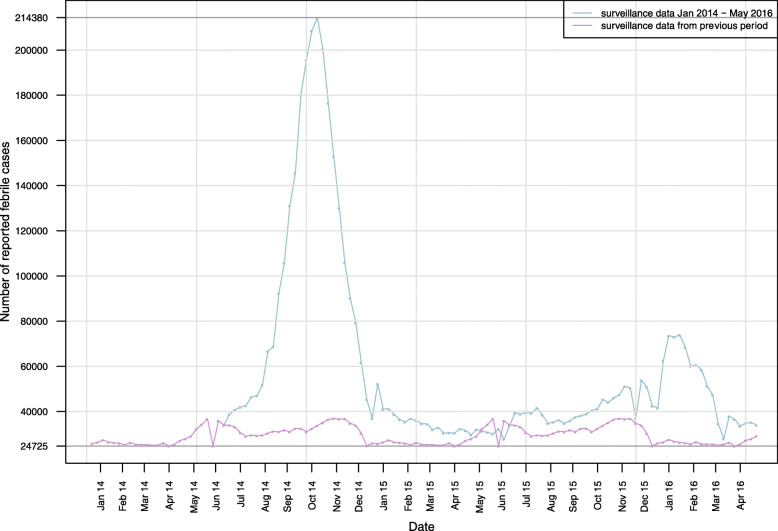
Weekly febrile case numbers reported between January 2014 and April 2016 and number of reported febrile cases from previous surveillance period

**Fig. 2:**
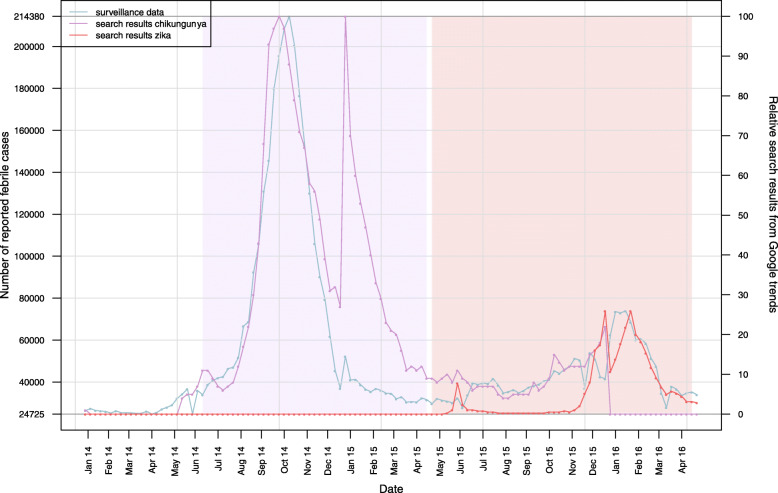
Line diagram depicting acute febrile case numbers reported and Google Trends queries for Chikungunya and Zika in Venezuela between January 2014 and May 2016. Purple and red shaded areas highlight case numbers attributed to Chikungunya and Zika virus, respectively, corresponding to the periods where each disease was circulating in the region. The point splitting the timeline corresponds with the initial Zika-related Google Trends query detected

Line graphs for officially reported febrile cases and Google Trends data for Chikungunya and Zika are presented in Figs. 1 and 2. The contrast between expected and observed acute fever cases, according to the MoH, is shown in Fig. 1. Time trends showed bi-modal distributions related to Chikungunya and Zika search queries, respectively. For Chikungunya, two peaks in Internet search queries between July 2014 and March 2015 can be identified. Similarly, two peaks in search queries related to Zika can be seen between November 2015 and March 2016. These peaks coincide with officially reported acute fever cases in the same periods (Fig. 2).

### Time-series cross-correlation

The linear association between disease surveillance and Google Trend patterns was assessed using cross-correlations, as shown in Table 1. Acute febrile cases occurring between 25th of June 2014 and 23rd of April 2015 were attributed to the Chikungunya virus, while cases occurring between 30th of April 2015 and 29th of April 2016 were ascribed to Zika virus. Cross-correlations were based on dates from these specific periods for each disease separately. Strongest correlations were shown at a lag of 0 (*r* = 0.784) for Chikungunya and + 1 (*r* = 0.754) for Zika, meaning that Google Search results for Zika depict the officially reported febrile cases best with a delay of 1 week.

**Table 1:** Bi-directional cross-correlation coefficients displaying the linear association between Google Trends records of Chikungunya and Zika and registered febrile cases in 2014–2016

Disease	Lag in weeks						
	−3	−2	−1	0	+ 1	+ 2	+ 3
**Chikungunya**	0.564	0.677	0.745	0.784	0.734	0.666	0.585
**Zika**	0.618	0.660	0.671	0.705	0.754	0.753	0.710

## Discussion

This study aimed to evaluate the usefulness of Google Trends as an indicator of the epidemic behaviors of Zika and Chikungunya in a context of sparse data availability. The analysis yielded high positive correlations between relative Google search volume for Zika- and Chikungunya-related search queries and official data on undiagnosed febrile cases. In the case of Zika, the highest correlation was observed when Google trends data were artificially shifted by a one-week lag.

In the present analysis, it was assumed that unexpected peaks of undiagnosed acute febrile cases corresponded to the Zika and Chikungunya incidence which the surveillance system was unable to reflect. Due to a lack of laboratory confirmation and official efforts to underestimate these outbreaks from 2013 to 2016 the Venezuelan MoH failed to report cases of both diseases. Thus, as the only official data available, undiagnosed acute febrile cases were used instead. To our knowledge, this is the first study that has compared web search queries and surrogates of Chikungunya and Zika cases. In light of the deteriorated surveillance system, sparse epidemiologic data, and striking peaks of acute fever without other feasible explanations this approach became a valuable option.

The time-series were normalized before cross-correlation analysis. This was performed in order to account for different units (relative search volume; number of cases). The time-series represent outbreak behaviors, which are processes with mean and variance/covariance that may vary in time. In particular, it is expected that there may be outbreaks of the infection during which more than the ‘usual number’ of cases are reported. For analysis, the sampled time intervals of both time-series were restricted to the same time periods to minimize the impact of non-stationarity on cross-correlation estimates.

The bimodal behavior of the search query data raises questions about the reasons for this unusual distribution. When assessed visually, the search data mostly correspond well to the accumulation of fever cases. However, in chikungunya-related searches, the second peak exceeds the amount of fever cases. Although it can be assumed that many searches are motivated by the health status of individuals and the need for health information, it cannot be excluded that other factors such as the media presence of both diseases are also partly responsible for the accumulation of Google searches.

Previous studies have demonstrated that internet search queries related to Zika and Chikungunya can provide good estimates of officially reported cases and can even be used to predict outbreak dynamcis [15, 27–29]. Moreover, Google Trends has also been shown to successfully estimate cases of other viral diseases such as dengue fever and influenza [30, 31]. However, on one side not only the affected individuals but also the public interest, fear or curiosity may generate online search queries during an outbreak leading to exaggerated estimates [32]. On the other side, it seems conceivable that once the public interest wanes, the number of searches may decline even if an outbreak is still ongoing. However, in this study this observation was not made. In the case of Chikungunya, the interest in Chikungunya expressed by Google searches increased while the alleged number of cases of the disease decreased.

The strong positive correlations demonstrated in the two time-series support the suitability of search queries used as a complementary tool for outbreak monitoring. One key advantage of this approach is that the data is readily available and freely accessible. Thus, it has the potential to contribute to the timely detection and public health response. Furthermore, harnessing web data for strengthening surveillance represents an additional cost-effective option.

Chikungunya and Zika virus are transmitted by the same vector. Currently, there is no specific treatment available against Zika or Chikungunya virus infections. Licensed vaccines are under development but not yet available [33, 34]. Preventive measures to combat both diseases as well as Dengue virus are based on similar vector control approaches and public education [1, 35, 36]. This tool could be useful for informing the design of vector control measures according to place and time of disease occurrence.

Nevertheless, there are some limitations to this study that need mentioning. It has been pointed out that Internet searching behavior related to a disease is susceptible to the impact of media and searches related to other diseases with similar symptoms (like other endemic arthropod-borne viruses) [37]. Thus, search patterns do not necessarily coincide with the actual disease dynamics but may also reflect curiosity, temporary awareness, and fear of a disease. Another important consideration is the regional variation in Internet penetration. The disease activity is likely to be underestimated in rural areas that are less likely to be served by Internet access.

Our models fit nationally aggregated search data with national case counts, making it useful for national surveillance purposes and decision-making. However, analyses based on finer spatial units would allow for more targeted interventions in areas with high disease burden.

Chikungunya, Zika, and Dengue virus infections share similar clinical characteristics. Fever is present in all three diseases [38]. This renders an incorrect self-diagnosis by web search information seekers likely. Furthermore, it is difficult to determine the share of undiagnosed febrile illness attributed to Chikungunya and Zika. In our study, the problem has been solved by a temporal distinction. However, acute fever due to other causes such as Dengue virus infections limits the accuracy of the results.

Despite the limitations of this tool, using Internet search queries might help to overcome some of the gaps that exist in Chikungunya and Zika surveillance in the Venezuelan context. Google Trends is not designed to replace traditional surveillance but to inform and complement it in the context of sparse epidemiologic data, absence of adequate surveillance, or censure.

## Conclusion

CDSSs in developing countries face problems providing data in a timely and reliable manner to facilitate an adequate response to infectious diseases. In the Venezuelan context, the CDSS is further challenged by the ongoing humanitarian crisis that has affected the health system severely. Unusual peaks of acute fever and Internet search queries related to Chikungunya and Zika could be used as proxy indicators of the actual outbreak activity. Especially in the context of sparse official data, Google search queries represent a valuable and timely indicator of disease activity.

## Data Availability

The Google Trends dataset generated for the current study is available and can be replicated at https://trends.google.com/trends/ The complete weekly epidemiological report of the Venezuelan MoH used for this study is currently not available to the public. In the last attempt to access the data, online versions of the bulletins until the year 2013 could be found: https://drive.google.com/drive/folders/0By6RZhEqt4ajUjFEeDg5dnVsVTQ/

## Abbreviations

CDSS: Communicable Disease Surveillance System
MoH: Ministry of Health
PAHO: Pan American Health Organization
WHO: World Health Organization
